# Adequacy of alcohol-based handrub solution production practice in response to COVID-19 in public hospitals found in Addis Ababa, Ethiopia: a multicentered cross-sectional study

**DOI:** 10.1186/s40545-021-00321-y

**Published:** 2021-05-02

**Authors:** Muluken Nigatu Selam, Regasa Bayisa, Andualem Ababu, Mahdi Abdella, Edessa Diriba, Minychel Wale, Assefa Mulu Baye

**Affiliations:** 1 Department of Pharmaceutics and Social Pharmacy, School of Pharmacy, College of Health Sciences, Addis Ababa University, P.O. Box 1176, Addis Ababa, Ethiopia; 2 Pharmaceutical and Medical Equipment Directorate (PMED), Ministry of Health, Addis Ababa, Ethiopia; 3 All African Leprosy, Tuberculosis Rehabilitation and Training Centre (ALERT), Addis Ababa, Ethiopia; 4 Department of Pharmacology and Clinical Pharmacy, School of Pharmacy, College of Health Sciences, Addis Ababa University, Addis Ababa, Ethiopia

**Keywords:** ABHR, COVID-19, Compounding, Hospitals, Quality, Documentation

## Abstract

**Background:**

Proper hand hygiene using alcohol-based handrub (ABHR) is an effective preventive approach for the current Coronavirus Disease 2019 (COVID-19) pandemic and other infections. World Health Organization recommends local production of ABHR solution in healthcare settings which provides a feasible alternative to the use of relatively expensive commercially produced hand sanitizers. The aim of this study was to explore the adequacy of ABHR solution production practice in response to COVID-19 in public hospitals of Addis Ababa, Ethiopia.

**Methods:**

A cross-sectional observational study was applied using assessment checklist for evaluation of the adequacy of ABHR production practice in compounding units of public hospitals. The evaluation was done with regard to the standard requirements as per the checklist. Statistical Package for Social Sciences (SPSS) version 23 was used for data entry and analysis. Descriptive statistics was employed for analyses of data and categorical variables were described by frequencies and percentages.

**Results:**

Out of the 13 public hospitals observed in the study, 11 facilities had dedicated premises for compounding of ABHR solution. Seven facilities determined the concentration of ethanol in ABHR solution using alcoholmeters. Only one health facility had a titration kit and performed a strength test for the hydrogen peroxide raw material. Thermal and chemical disinfection processes were practiced for cleaning of recycled dispensing bottles only in 3 and 2 hospitals, respectively. Most of the hospitals (11 facilities) had standard operating procedures (SOPs) for production, but the majority lack SOPs for beyond-use-date assignment (11 facilities), premise and equipment cleaning (12 facilities), and disinfection of recycled bottles (12 facilities).

**Conclusion:**

Most hospitals have fulfilled the majority requirements of premises required for compounding of ABHR solution in their facilities. Five hospitals did not verify the concentration of ethanol in the ABHR solution which might affect the effectiveness of the product. Generally, lower compliance of the majority studied hospitals to good compounding practice was observed during ABHR solution production especially for product preparation, quality control, and documentation.

**Supplementary Information:**

The online version contains supplementary material available at 10.1186/s40545-021-00321-y.

## Introduction

The emergence of the COVID-19 (Coronavirus Disease-2019) pandemic has risen to be a significant global public health concern. Globally, a total of 80,228,249 confirmed COVID-19 cases, and 1,756,998 deaths were reported affecting over 200 countries as of 25 December 2020. It is an infectious disease caused by severe acute respiratory syndrome coronavirus 2 (SARS-CoV-2) [1, 2]. On March 2020, Ethiopia registered the first Coronavirus Disease-2019 (COVID-19) case [3]. As of December 25, 2020, Ethiopia has reported a total of 121,880 confirmed COVID-19 cases and 1,897 deaths [1].

Among the suggested measures for prevention of the rapidly spreading COVID-19 and other healthcare-associated infections (HCAIs), hand hygiene is the simplest, effective, and least expensive one. Given the dangers imposed by this disease, proper hand hygiene through handwashing or use of hand sanitizer has been emphasized as it is the cornerstone of good infection prevention and control practice [4–7]. Choosing the appropriate method of hand decontamination is necessary which depends on several factors. In many developing countries where access to handwashing facilities is limited due to a shortage of infrastructure and insufficient water sources, alcohol-based hand rub (ABHR) offers a viable alternative [8, 9].

Using ABHR for hand hygiene in healthcare settings is recommended by the World Health Organization (WHO) because of the broad antimicrobial spectrum (including SARS-CoV-2), rapid antimicrobial effect, easy availability at the point of care, good skin tolerance, and general acceptability to health professionals [5, 10–12]. Most effective alcohol-based formulations contain 60% to 95% of alcohol that denatures microbial proteins and inactivates viruses [6, 13]. Evidences show that the introduction of ABHR is associated with a higher hand-hygiene compliance rate [14–16]. However, problems of availability and affordability limit the use of such ABHR products in low- and middle-income countries (LMICs) [17, 18]. Therefore, local production of WHO-recommended ABHR formulations provides a feasible alternative to the use of relatively expensive, commercially produced ABHR products, particularly for LMICs [6, 8, 19]. A cost analysis from a case study conducted in Rwanda revealed a 71% financial savings when producing in-house ABHR rather than a commercially bought sanitizer [7]. Promoted globally by the WHO since 2010, local production has been adopted in some healthcare facilities as the preferred means of providing ABHR for their staff at the point of care [20–23]. Due to the contagious nature of COVID-19, extensive use of hand disinfectants is observed globally for the prevention of its transmission [2].

To handle the spread of COVID-19, urgent and collaborative work is required by countries especially from healthcare organizations as the pandemic severely affected the supply chain including hand sanitizers availability [24, 25]. Ethiopia is capacitating the healthcare system since the COVID-19 pandemics [26]. Initiated by the Ethiopian Ministry of Health, some hospitals in the country have started the production of ABHR solution in response to COVID-19 to meet their demand and beyond.

The process of facility-based ABHR production is quite simple and the WHO guide to local ABHR production provides a clear outline of materials required, methodology, and recommendations for the best outcome [20]. While the preparation of ABHR solution seems simple, understanding the basics of compounding non-sterile preparations and adhering to good compounding practice (GCP) is important to produce safe, effective and quality products capable of preventing the transmission of COVID-19 and other HCAIs.

Even though the in-house production of ABHR benefits the health facilities from the consistent supply point of view, such practice is challenged with a lack of the production expertise, equipment needed to assure quality control, and finance for procuring the needed amount of raw materials [9].

The compounding of ABHR in healthcare facilities should be practiced in a manner that keeps product quality and safety of compounding personnel. Noncompliance with the standard of practice during ABHR production may have undesirable consequences such as compromising product efficacy and exposing the compounding personnel at risk. Evaluation of the adherence of ABHR production practice to standard requirements for the preparation of non-sterile products is suggested [21]. So, the current observational study aimed at evaluation of the adequacy of ABHR solution production practice at public hospitals in reference to the requirements.

## Methods

### Study setting and period

This study was conducted from May 11–26, 2020 in all 13 public hospitals found in Addis Ababa, Ethiopia. The list of hospitals included in this study is indicated in Table 1.

**Table 1:** List of public hospitals included in the study

S.N.	Name of the hospitals	S.N.	Name of the hospitals
1	Tikur Anbessa Specialized Hospital (TASH)	8	Tirunesh Beijing Hospital (TBH)
2	St Paul Hospital Millennium Medical College (SPHMMC)	9	Gandhi Memorial Hospital (GMH)
3	All African Leprosy, Tuberculosis Rehabilitation and Training Centre Hospital (ALERTH)	10	Zewditu Memorial Hospital (ZMH)
4	Eka Kotebe General Hospital (EKGH)	11	Addis Ababa Burn, Emergency and Trauma Hospital (AABETH)
5	St Peter Specialized Hospital (SPSH)	12	Amanuel Mental Specialized Hospital (AMSH)
6	Minellik II Referral Hospital (MRH)	13	Ras Desta Damtew Memorial Hospital (RDDMH)
7	Yekatit 12 Hospital Medical College (YHMC)		

*ABHR* alcohol-based handrub

### Study design

A single-time, unannounced observation-based cross-sectional study was used. The assessment checklist was developed by adopting different standard sources [20, 27–33].

### Eligibility criteria

Public hospitals that started ABHR solution production were included in the study.

### Sample size and sampling technique

A purposive sampling technique was used. All public hospitals having ABHR solution production unit were included in the study to assess the adequacy of their ABHR solution production practice.

### Data collection instrument

The ABHR production units of the hospitals were observed to evaluate the adequacy ABHR solutions production practice to the standard requirements as per the checklist (Additional file 1) which was developed following standard guidelines including WHO, Ministry of Health-Ethiopia SOP for ABHR solution production and others [20, 27–33].

The observation checklist consisted of closed-ended items on the adequacy of compounding and storage premises, the compounding process, quality control (QC) activities, hygiene and sanitation; and activities recording and reporting system.

### Data collection process and quality assurance

The observers were senior pharmacists trained by expert pharmacists. The selection of data collectors was based on the educational level and experience in ABHR solution production. The training was given to 6 data collectors and 2 supervisors about the objectives of the study and the process of the data collection for 2 days. Strict supervision by the principal investigators was conducted during data collection; meanwhile, any doubts in the checklist were clarified. A pretest was conducted at Adama Hospital Medical College.

### Data analysis

Before entry, data were coded, checked for completeness, and accuracy. Then the data were entered and analyzed using Statistical Package for Social Sciences (SPSS) version 23. Descriptive statistics was employed for analyses of data. Categorical variables were described by frequencies and percentages.

## Results

Among the 13 public hospitals in Addis Ababa considered for this survey, 12 were observed at the time of ABHR solution production and the production was interrupted in one hospital.

### Compounding premise

Of the public hospitals included in the study, 11 had dedicated premises for compounding of ABHR solution and other hospital-based non-sterile preparations like dermatological products (Table 2). Regarding the suitability of the compounding premises, most were properly designed in terms of location (11 hospitals), space (11 hospitals), and room ventilation (12 hospitals).

**Table 2:** Adequacy of compounding premise for the production of ABHR solution in study hospitals

Parameter	Observation	
	Yes (%)	No (%)
Dedicated compounding room is available	11 (84.6%)	2 (15.4%)
Compounding room is properly located	11 (84.6%)	2 (15.4%)
Compounding room is well ventilated	12 (92.3%)	1 (7.7%)
Compounding room has adequate light	10 (76.9%)	3 (23.1%)
Compounding room is protected from direct sunlight	11 (84.6%)	2 (15.4%)
Compounding room has enough space	11 (84.6%)	2 (15.4%)

*ABHR* alcohol-based handrub

### Compounding process

Twelve hospitals were engaged in the production of ABHR solution during the period of observation. Compounding personnel in some facilities did not check the expiry dates (4 hospitals) and strength (4 hospitals) of the starting chemicals from the labels before commencing the mixing operations (Table 3). To the actual compounding of ABHR solutions, the order of ingredients’ incorporation into the mixing vessel was properly followed by the majority hospitals (10 facilities), but only compounding personnel in three hospitals were mixing the solutions in divided doses.

**Table 3:** Compounding activities during the production of ABHR solution in study hospitals

Compounding activity/process	Observation	
	Yes (%)	No (%)
The expiry dates of raw materials are checked	8 (66.7%)	4 (33.3%)
The strength of raw materials is checked	8 (66.7%)	4 (33.3%)
The quantities of each ingredient are calculated	10 (83.3%)	2 (16.7%)
The quantity of each ingredient is measured	11 (91.7%)	1 (8.3%)
Mixing order is proper	10 (83.3%)	2 (16.7%)
Mixing is done in divided dose	3 (25.0%)	9 (75.0%)
Compounding process is online	6 (50.0%)	6 (50.0%)

*ABHR* alcohol-based handrub

### Quality control

Six of the 13 facilities had alcoholmeters to evaluate the concentration of alcohol at the study facilities. One health facility had a titration kit to determine the strength of hydrogen peroxide (H_2_O_2_). Table 4 shows the different quality control activities performed in 12 of the survey hospitals by excluding TBH where the ABHR solution production was interrupted during the study period. The alcohol strength was determined in six hospitals for the starting (raw) ethanol and in 7 hospitals for the ABHR solution. Only one hospital performed H_2_O_2_ raw material strength determination but none of the hospitals were determining the strength of H_2_O_2_ for the ABHR solution. Other observed quality control activities include a physical inspection of the ABHR solution (7 hospitals), checking the integrity of packaging (7 hospitals), and checking the legibility of labeling information and its comprehensiveness (7 hospitals).

**Table 4:** Adequacy of quality control activities for the production of ABHR solution in study hospitals

Quality control activities	Observation	
	Yes (%)	No (%)
Raw ethanol is tested for strength	6 (50.0%)	6 (50.0%)
Ethanol strength for the ABHR solution is determined	7 (58.3%)	5 (41.7%)
Strength for H_2_O_2_ raw material is determined	1 (8.3%)	11 (91.7%)
ABHR solution is tested for H_2_O_2_ strength	0 (0.0%)	12 (100.0%)
ABHR solution is physically inspected	7 (58.3%)	5 (41.7%)
ABHR solution dispenser is checked for the integrity of packaging	7 (58.3%)	5(41.7%)
ABHR solution dispenser label is checked for its legibility and comprehensiveness of information	7 (58.3%)	5 (41.7%)

*ABHR* alcohol-based handrub

### Storage premise and condition

A separate storage room was available for raw materials (6 hospitals) and the ready-to-use ABHR solution (7 hospitals). In the majority of hospitals these raw materials were stored in cool and dry places (11 facilities) and in a way protected from direct sunlight (12 facilities). In 11 of the observed hospitals the bottles filled with ABHR solution were quarantined for 72 h before dispatch. The details of storage premise and condition are indicated in Table 5.

**Table 5:** Adequacy of storage premises and condition for the production of ABHR solution in study hospitals

Parameter	Observation	
	Yes (%)	No (%)
Separate storage room is available for raw materials	6 (46.2%)	7 (53.8%)
Raw materials are stored in cool and dry place	11 (84.6%)	2 (15.4%)
Raw materials are stored in a way protected from direct sunlight	12 (92.3%)	1 (7.7%)
Separate storage room is available for the finished ABHR products	7 (53.8%)	6 (46.2%)
ABHR solution is stored in cool and dry place	12 (92.3%)	1 (7.7%)
Finished products are stored in a way protected from direct sunlight	11 (84.6%)	2 (15.4%)
Ready-to-use ABHR solution is quarantined for 72 h before dispatched	11(84.6%)	2 (15.4%)

*ABHR* alcohol-based handrub

### Hygiene and sanitation

The compounding personnel hygienic conditions and sanitation measures were observed in those hospitals compounding ABHR solution during the survey (12 facilities) (Table 6). Handwashing was practiced before starting the compounding process in 8 hospitals. Donning of the required personal protective equipment by compounding personnel was observed in 10 hospitals. Before and after the production of the ABHR solution, the compounding area and premise were properly cleaned in 11 hospitals, and production equipment were cleaned in 9 hospitals. All health facilities reused the bottles for dispensing of the product. The dispensing bottles were refilled after thermally disinfected (3 hospitals) and chemical disinfection processes (2 hospitals).

**Table 6:** Personal hygiene and sanitation practice during ABHR solution production and quality control in study hospitals

Parameter	Observation	
	Yes (%)	No (%)
Personnel washed their hands before starting the compounding process	8 (69.2%)	4 (30.8%)
There is proper attire system during production	10 (84.6%)	2 (15.4%)
There is proper attire system during QC (*N* = 9)	5 (55.5%)	4 (45.5%)
Compounding area is cleaned properly before and after operation	11 (92.3%)	1 (7.7%)
There is dust bin for holding wastage materials (gloves, face mask, etc.)	12 (100%)	0 (0.0%)
All production and QC equipment are cleaned after use	9 (76.9%)	3 (23.1%)
Recycle packaging bottles are cleaned by simple washing of empty bottles	6 (50.0%)	6(50.0%)
Recycle packaging bottles are thermally disinfected	3 (25.0%)	9 (75.0%)
Recycle packaging bottles are cleaned by chemical disinfection	2 (15.4%)	10 (84.6%)

*ABHR* alcohol-based handrub, *QC* quality control

### Documentation

Most of the hospitals had standard operating procedures (SOPs) for production (11 facilities) and distribution records for the dispensed products (11 facilities). Among the least available documents in the health facilities were beyond-use-date (BUD) assigning SOP (2 facilities), safety and precaution measures guidelines (2 facilities), SOP for premise and equipment cleaning (1 facility), and SOP for disinfection of recycled bottles (1 facility). The availability of documents for ABHR solution production and QC is indicated in Table 7.

**Table 7:** Documents availability for ABHR solution production in study hospitals

Document type	Observation	
	Available (%)	Not available (%)
SOP for production	11 (84.6%)	2 (15.4%)
Master formulation record (MFR)	5 (38.5%)	8 (61.5%)
SOP for QC operation	4 (30.8%)	9 (69.2%)
SOP for assigning of BUD	2 (15.4%)	11 (84.6%)
SOP for cleaning of premises and equipment	1 (7.7%)	12 (92.3%)
SOP for disinfection of recycled bottles	1 (7.7%)	12 (92.3%)
Distribution records for ABHR solution	11 (84.6%)	2 (15.4%)
Guidelines for safety and precaution measures	2 (15.4%)	11 (84.6%)

*ABHR* alcohol-based handrub, *QC* quality control

### Comparison of hospitals for documentation and quality control activities

**Fig. 1:**
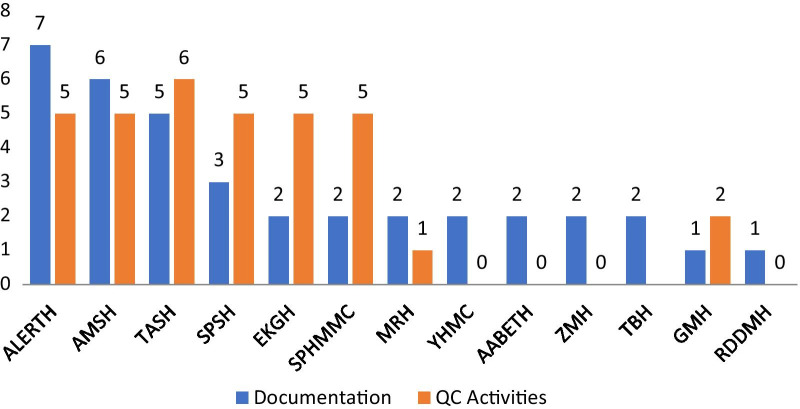
Public hospitals’ total score on documentation and quality control activities for ABHR solution production practice. *ABHR* alcohol-based handrub, *AABETH* Addis Ababa Burn, Emergency and Trauma Hospital, *ALERTH* All African Leprosy, Tuberculosis Rehabilitation and Training Centre Hospital, *AMSH* Amanuel Mental Specialized Hospital, *EKGH* Eka Kotebe General Hospital, *GMH* Gandhi Memorial Hospital, *MRH* Minellik II referral hospital, *QC* quality control, *RDDMH* Ras Desta Damtew Memorial Hospital, *SPHMMC* St Paul Hospital Millennium Medical College, *SPSH* St Peter Specialized Hospital, *TASH* Tikur Anbessa Specialized Hospital, *TBH* Tirunesh Beijing Hospital, *YHMC* Yekatit 12 Hospital Medical College, *ZMH* Zewditu Memorial Hospital

Comparison among the studied hospitals was made for document availability and QC activities during ABHR production (Fig. 1). TASH had scored the maximum QC activities for the evaluated parameters (scored 6/7) whereas YHMC, AABETH, ZMH, and RDDMH had fulfilled none of the activities (scored 0/7). Concerning document availability, ALERTH had majority of them (7/8) compared to others and GMH and RDDMH had scored the minimum value (1/8).

## Discussion

All hospitals were compounding ABHR solution as per one of the WHO formulation, which contains 80% v/v ethanol as an active agent and H_2_O_2_, glycerol, and water are the other ingredients [20].

One of the key requirements for compounding services in health facilities is the establishment of a dedicated premise. The compounding room should have adequate space and equipment. Additionally, compounding rooms should be designed in such a way to have proper ventilation, light, and protection from direct sunlight to preserve products and provide comfort for operators [28–30]. ABHR should be prepared in a room fulfilling the above requirements. From the current study, it was observed that most hospitals (11 facilities) had premises dedicated to compounding of non-sterile preparations including ABHR solutions and the rest were working in temporarily provided rooms.

The site of compounding rooms in the majority hospitals (84.6%) was found to be appropriate since it is not closer to high traffic area rooms (like outpatient department, patient waiting rooms, etc.) and away from sources of ignition (like heat sources and electric motors). Such a proper location is recommended for preparing good quality products and protected from contamination of any type and accidental fire hazard since ethanol is flammable [30, 33]. Regarding space, the majority hospitals (84.6%) had met the minimum 9 m^2^ floor space set as a requirement by Ethiopian Food and Drug Authority (EFDA) (formerly known as Drug Administration and Control Authority (DACA)) for compounding rooms [27]. Most hospitals’ compounding premises were also found to be well ventilated (92.3%) through a sufficient number of windows, well lighted (76.9%), and protect materials from undue exposure to direct sunlight (84.6%). Such building requirements for compounding premises are also indicated in different sources [28–30]. Few hospitals shall make their compounding facilities suit for its purpose by taking measures starting from minor actions of covering of windows to protect rooms from direct sunlight entrance and installing lights up to a major action of changing the premise.

WHO has created relatively simple formulation recipes for the production of ABHR solution in healthcare facilities especially found in LMICs [20]. Despite this, the production should be done as per GCP to make safe, effective, and quality products. Before starting the mixing of ingredients, the raw ingredients should be checked for their identity, labeled strength, and shelf life. Such preparatory stages of compounding are desired for the prevention of unintentional use of expired or different chemicals that may affect the health of users or compounding personnel since some chemicals are incompatible and toxic. At the time of observation, compounding personnel in the majority hospitals were checking the labels of H_2_O_2_ and glycerol containers for strength (66.7%) and shelf life (66.7%) before measuring the required quantity for compounding. Mix-ups of chemicals can occur especially in facilities where compounding of other types of preparations are in place. Regardless of the variety of preparations, careful checking of raw materials’ labels should be a habit of compounding personnel for every batch. In all hospitals, ethanol was taken from large containers (capacity of ≥ 500 L) with claimed strength of 96% v/v but not labeled.

Compounding personnel in most facilities (91.7%) measured the required amount of ingredients using measuring cylinders and beakers after taking the initial strengths of ingredients and final volume of ABHR solution into consideration to prepare products as per WHO’s recommendation [20]. In one facility, the glycerol and H_2_O_2_ was added to the mixing container by noting their containers labeled quantity but not measured. Measuring the required quantity of each ingredient is required as there may be a variation of fill volume from containers of chemicals. The mixing of ingredients shall be in such a way that glycerol is added in between other ingredients’ addition. This is applied by considering the viscous nature of glycerol to get all ingredients mixed easily. Compounding personnel in most study facilities (83.3%) mixed ingredients following the recommended mixing order [20]. Although the ingredients used to prepare the ABHR solution are miscible with one another, they should be incorporated portion by portion in multistage with vigorous mixing between each addition to ensure a homogeneous mixture [34], but this was practiced in few hospitals (25.0%). Compounding personnel in half of the hospitals (50.0%) were conducting activities of ABHR preparations in a logical order. This was done by assigning spaces in the compounding room and carrying out activities (measuring of ingredients, mixing, filling, labeling, etc.) in a sequential and orderly manner that avoids crowding of table’s space with equipment, chemicals, packaging, and labeling materials. Such orderly placement and activities are important to avoid congestion, to minimize the risks of confusion and contamination, and to prevent mix-ups among components, containers, labels, in-process materials, and finished products [29].

Quality assurance and quality control tests are necessary to ensure that high-quality products are prepared consistently [29]. Assuring the quality of ABHR solution is necessary to guarantee its effectiveness for infection prevention including the current pandemic COVID-19**.** Every production batch should be tested for the strength of ethanol, which is an active agent of the ABHR solution and claimed to be 80% v/v [9, 20]. Since there is a clear positive association between the extent of microbial reduction and the concentration of alcohol contained in ABHR products, verification of ethanol strength for all batches is recommended [11] and this test was carried out in 58.3% of the studied hospitals. Determination of H_2_O_2_ concentration is the other quality control test recommended for the solution if possible [9, 20]. H_2_O_2_ strength of the starting material was verified only in a single hospital by redox titration but none of them confirmed the ABHR products for this test. A similar finding was indicated from another assessment study [10]. Lack of equipment at the facilities hampered such QC tests. On the other hand, for the rational use of ABHR, it is recommended to place a good readable sign on the dispenser indicating the direction to use, precautions, shelf life, etc. [35]. In this respect, only compounding personnel in about half of the facilities (58.3%) checked labels on dispensers.

Raw materials for non-sterile preparations and finished products should be stored and kept safely under conditions that will preserve their quality and purity [28]. Maintaining good storage conditions for ABHR products is desired due to the volatile nature of alcohol. Products in the majority hospitals were stored properly and met the requirements [20, 29]. Besides, most hospitals (84.6%) stored their products for 3 days, mainly in bulk containers, before dispatching to individuals and wards. Such placing of ABHR solution in quarantine allows time for any spores present in the product to be destroyed [20].

Good personal hygiene and sanitation were observed in most hospitals during compounding of the ABHR solution. Adherence to standards of cleaning, personal hygiene, and protective clothing is important during compounding for safe handling of chemicals and protection of products from contamination [28]. Dispensing bottles were reused for filling and distribution of the ABHR product in all hospitals. Thermal or chemical disinfection of recycled bottles shall be in place in health facilities before the refilling of the bottles for delivering effective ABHR solution since reprocessing of empty dispensers by simple washing may lead to handrub contamination [10, 20]. But such practices were not observed in the majority of the study facilities and a similar result was reported in another study [10]. The reasons could be a lack of awareness on the importance of bottles’ disinfection process and the absence of required supportive staff, cleaning equipment or disinfection guideline. The fear of handling recycled bottles because of the highly contagious coronavirus might be the other reason restricting peoples from disinfecting bottles.

Facilities, where non-sterile products are prepared, must have and maintain written or electronic documentation to demonstrate compliance with the requirements [29]. The document types obtained during the study period in the majority hospitals were only distribution records (84.6%) and SOP for the production of ABHR solution (84.6%). Although documentation is the key element of GCP, most facilities did not have most of the relevant documents for the production of ABHR and other non-sterile preparation. Facilities compounding non-sterile preparation must develop SOPs on all aspects of the compounding operation [29]. Absence of such documents might contribute to the poor adherence of facilities towards some activities (eg QC and disinfection of recycled bottles) required during ABHR production.

From the combined total score for documentation and QC activities (15 scores), ALERT hospital had fulfilled the maximum parameters (scored 15) followed by AMSH and TASH which can be taken as models for other hospitals. On the other hand, minimum scores were obtained for RDDMH (1/15), YHMC (2/15), AABETH (2/15), ZMH (2/15), and TBH (2/15) that showed a need for much improvement by them concerning the observed documentation and QC parameters. Such comparison among hospitals helped some of them for improving their ABHR production practice by sharing experiences from those hospitals that met the majority requirements.

Such an observational study is believed to be better than the self-reported for assessing the ABHR compounding practice conformity to the requirements. This study is the first in its kind, to our experience, and it can be used as a baseline for future similar studies. Despite this, the study has some limitations. Although there are many health facilities in the city, private hospitals, and public health facilities other than hospitals were not considered. Moreover, a single-time observational study design was used, making it difficult in generalizing the status of hospitals regarding ABHR solution production practice.

## Conclusion

Proper hand hygiene using hand sanitizer plays a significant role in combating COVID-19 disease. While the ABHR production practice in health facilities is recommended during the COVID-19 crisis, the quality of the product should be assured especially for its alcohol content. Five hospitals did not verify the concentration of ethanol in the ABHR solution, which might affect the quality and effectiveness of the product. Most hospitals have fulfilled the majority requirements of the premise and equipment required for compounding of ABHR solution in their facilities. All health facilities reused the bottles for dispensing of the product, but the majority of them did not perform the recommended chemical (84.6%) and thermal (75.0%) disinfection. The documentation system in most hospitals was found to be inadequate which needs to be improved. Generally, lower compliance of studied hospitals to GCP was observed during ABHR solution production especially regarding product preparation, QC, and documentation.

## Recommendations

Ministry of Health-Ethiopia shall continuously monitor and evaluate the health facilities for their ABHR solution production practice and provide technical support when needed. Also, hospitals should undertake self-evaluation of their ABHR solution production practice and make appropriate correction whenever necessary. The researchers also recommended that interventional studies shall be carried out on the adequacy of ABHR solution production in the country’s health facilities which already started the production. Furthermore, the drug regulatory body of the country should inspect health facilities engaged in the compounding of ABHR products and other non-sterile preparations for their adherence towards the local and international standards.

## Supplementary Information

**Additional file 1.** Checklist.

## Data Availability

All data generated or analyzed during this study are included in the manuscript and Additional file 1.
