# Supplementary material for: Adequacy of alcohol-based handrub solution production practice in response to COVID-19 in public hospitals found in Addis Ababa, Ethiopia: a multicentered cross-sectional study
Source: J Pharm Policy Pract. 2021 May 2;14:39. doi: 10.1186/s40545-021-00321-y (PMC8088824; doi:10.1186/s40545-021-00321-y)
Supplement: Supplementary file 1 — Additional file 1. Checklist. [file 40545_2021_321_MOESM1_ESM.docx]

## Annex: Checklist

**Instruction for the checklist**

Please complete the checklist by writing the following numbers in the ‘***observation’*** column:

0 - **No or Not available**

1 - **Yes or available**

2 - **Not applicable**

If you have any additional information, write in the ‘***remark***’ column.

Name of the hospital___________________________________________

| 1. **Compounding premise** | | | | |
| --- | --- | --- | --- | --- |
| **S.N** | **Item** | **Description** | **Observation** | **Remark** |
| 1.1 | Is there dedicated compounding room? | Specifically allocated for compounding |  |  |
| 1.2 | Is the location of compounding room appropriate? | Away from source of contamination and explosion (e.g. wards, bacteriological laboratory, kitchen, waste disposal site, electric motors etc) |  |  |
| 1.3 | Is the compounding room well ventilated? | Adequate windows and ventilators |  |  |
| 1.4 | Has the compounding room adequate light? | Light that enables proper production and packaging |  |  |
| 1.5 | Is the compounding room protected from direct sunlight? |  |  |  |
| 1.6 | Has the compounding room enough space? | - At least 3m X 3m = 9 m^2^ - Avoid errors and difficulty of measuring, mixing etc - Allow free movement of operators |  |  |
| 1. **Compounding process** | | | | |
| 2.1 | Is there checking of the expiry dates of raw materials? |  |  |  |
| 2.2 | Is there checking of the strength of raw materials? |  |  |  |
| 2.3 | Is there calculation of the amounts of each ingredient required for production of the ABHR solution? | This depends on the strength of starting materials and the intended ABHR solution to be produced? |  |  |
| 2.4 | Was the quantity of each ingredient measured? | Using appropriate beakers or measuring cylinders |  |  |
| 2.5 | Is the mixing order proper? | - Adding glycerol in between of alcohol or hydrogen peroxide addition - Adjusting of the final volume with water or alcohol |  |  |
| 2.6 | Is the mixing done in divided dose? | 2-3 shaking upon addition of ingredients |  |  |
| 2.7 | Is the process online?  (Is the space orderly arranged with proper placement of equipment and materials) | Room area/space classification in logical order according to GCP (Measuring→ mixing → packaging → labeling) |  |  |
| 1. **QC equipment availability** | | | | |
| 3.1 | Alcoholmeter | Alcoholmeter or hydrometer |  |  |
| 3.2 | H_2_O_2_ strip | Indicating H_2_O_2_ strength using specific color band |  |  |
| 3.3 | Titration kit | Indicating H_2_O_2_ strength by redox titration |  |  |
| 1. **QC activities** | | | | |
| 4.1 | Is strength of alcohol raw material determined? |  |  |  |
| 4.2 | Is the alcohol strength for the ABHR solution determined? |  |  |  |
| 4.3 | Is the strength for H_2_O_2_ raw material determined? |  |  |  |
| 4.4 | Is the H_2_O_2_ strength for the ABHR solution determined? |  |  |  |
| 4.5 | Is physical inspection of the ABHR solution carried out? | For any color and odor change or visible particulate matter |  |  |
| 4.6 | Is ABHR solution dispenser checked for the integrity of packaging? | To check its leak proof nature |  |  |
| 4.7 | Is ABHR solution dispenser label checked for its legibility and comprehensiveness of information? | - Product name with strength, - direction to use, - precaution, - BUD and - batch number |  |  |
| 1. **Storage premise and condition** | | | | |
| 5.1 | Is there a separate storage room for raw materials? |  |  |  |
| 5.2 | Do raw materials stored in cool and dry place? |  |  |  |
| 5.3 | Is there a separate storage room for finished products? |  |  |  |
| 5.4 | Do compounded ABHR solution stored in cool and dry place? |  |  |  |
| 5.5 | Do raw materials stored in a way protected from direct sunlight? |  |  |  |
| 5.6 | Do finished products stored in a way protected from direct sunlight? |  |  |  |
| 5.7 | Do compounded ABHR solution stored/quarantine for 72 hrs before dispatched? | Check distribution record |  |  |
| 1. **Hygiene and sanitation** | | | | |
| 6.1 | Does the operator practiced hand washing before starting the compounding process? |  |  |  |
| 6.2 | Is there proper attire system by compounding personnel during production? | Donning of all the necessary PPE (gown, glove, face mask, and eye goggle) |  |  |
| 6.3 | Is there proper attire system during quality control activities? | Donning of all the necessary PPE (gown, glove, face mask, eye goggle) |  |  |
| 6.4 | Is there proper cleaning of the compounding area before and after operation? |  |  |  |
| 6.5 | Are there dust bins for holding wastage materials (gloves, face mask, etc)? |  |  |  |
| 6.6 | Are all production and quality control equipment cleaned after use? |  |  |  |
| 6.7 | Are recycle packaging bottles cleaned by simple washing of empty bottles? |  |  |  |
| 6.8 | Are recycle packaging bottles thermally disinfected? | by submerging used empty bottles in boiling water for 20 min |  |  |
| 6.9 | Are recycle packaging bottles cleaned by chemical disinfection? | soaking the bottles in a chlorine solution (1000 ppm) for 15 min |  |  |
| 1. **Documentation** | | | | |
| 7.1 | Is there SOP for ABHR solution production? |  |  |  |
| 7.2 | Is there Master formulation record (MFR)? |  |  |  |
| 7.3 | Is there SOP for ABHR solution QC operation? |  |  |  |
| 7.4 | Is there SOP for assigning beyond use date (BUD) for ABHR solution? |  |  |  |
| 7.5 | Is there SOP for cleaning of premises and equipment? |  |  |  |
| 7.6 | Is there SOP for disinfection of recycled bottles? |  |  |  |
| 7.7 | Is there a document regarding the distribution of ABHR solution? |  |  |  |
| 7.8 | Is there document on safety and precaution measures? |  |  |  |
|  | | | | |
